# Abnormal Ulnar Variance: A New Perspective in Rheumatoid Arthritis-Related Joint Damage

**DOI:** 10.5152/ArchRheumatol.2025.11108

**Published:** 2025-06-23

**Authors:** Sertaç Ketenci, Mete Pekdiker, Bora Uzuner, Dilek Durmuş

**Affiliations:** 1 Department of Rheumatology, Ondokuz Mayıs University, Samsun, Türkiye; 2 Department of Rheumatology, Hatay Mustafa Kemal University, Hatay, Türkiye; 3 Department of Physical Medicine and Rehabilitation, Ondokuz Mayıs University, Samsun, Türkiye

**Keywords:** Pain, rheumatoid arthritis, ulnar variance

## Abstract

**Background/Aims:**

Rheumatoid arthritis (RA) is characterized by significant inflammation and joint damage. This study aims to investigate the frequency of abnormal ulnar variance (AUV) in RA patients and its associated factors.

**Materials and Methods:**

A total of 108 established RA patients meeting the 2010 ACR/EULAR RA criteria were included. After exclusions, the study proceeded with 98 patients. Demographic, laboratory, and clinical data were recorded. X-rays of the wrists were taken in accordance with the literature, with the forearm in a neutral rotation, the elbow flexed at 90°, the shoulder abducted at 90°, and ulnar variance was assessed with Hulten’s method. A displacement of 1 mm or more of the ulna relative to the radius was defined as AUV.

**Results:**

The average age was 58.11 ± 12.05 years, with 82% being female. The mean disease duration was 175.16 ± 100.5 months, and the average diagnostic delay was 16.4 ± 11.18 months. Abnormal ulnar variance was present in 47.9% of patients. In patients with AUV, the average UV for the right hand was +2.24 mm, while the average for the left hand was +2.40 mm. When considering all RA cases, the average UV was +1.06 mm for the right hand and +1.09 mm for the left hand. In the multivariate analysis, RA-type joint involvement (RJI) and severe joint involvement (SJI) were identified as independent predictors of AUV.

**Conclusion:**

This study suggests that AUV may be an important finding in established RA. Future larger-scale and prospective studies are needed to elucidate the significance of AUV in RA cases.

**Main Points**

- Abnormal ulnar variance (AUV) was identified in 47.9% of rheumatoid arthritis (RA) patients, with 93.6% of those cases showing positive ulnar variance.
- RA patients with AUV had significantly longer disease duration and higher rates of RF and anti-CCP positivity, indicating a more aggressive disease course.
- Radiographic RA-type joint involvement (RJI) and severe joint involvement (SJI) were determined as independent predictive factors for AUV.
- The presence of AUV was associated with higher usage of biologic and targeted synthetic DMARDs.
- The study proposes that AUV may serve as a marker of cumulative joint damage in long-standing RA and warrants inclusion in radiographic assessments.

## Introduction

Rheumatoid arthritis (RA) is one of the most common inflammatory rheumatic diseases in the population. It is observed more frequently in women than in men, and its prevalence in Western countries is 0.5%.^1^ The primary target tissue in RA, which is a multisystemic disease developing on the basis of autoimmunity, is the synovial tissue in peripheral joints. One of the key pathological features of chronic synovitis in RA is its erosive nature. This process not only leads to bone and cartilage destruction within the joint but also affects surrounding structures, including tendons and ligaments, due to persistent inflammation.^2^

The characteristic clinical feature of RA is chronic symmetrical polyarthritis. Involvement of the small joints of the hands and feet is a very common finding. The wrist is one of the target joints, and wrist involvement occurs in a significant proportion of cases. The erosive course starts early in patients with RA; joint erosion occurs in 63% of cases at the end of the 3rd year, while this rate is 10% in the first 8 weeks.^3^

**Figure 1.**
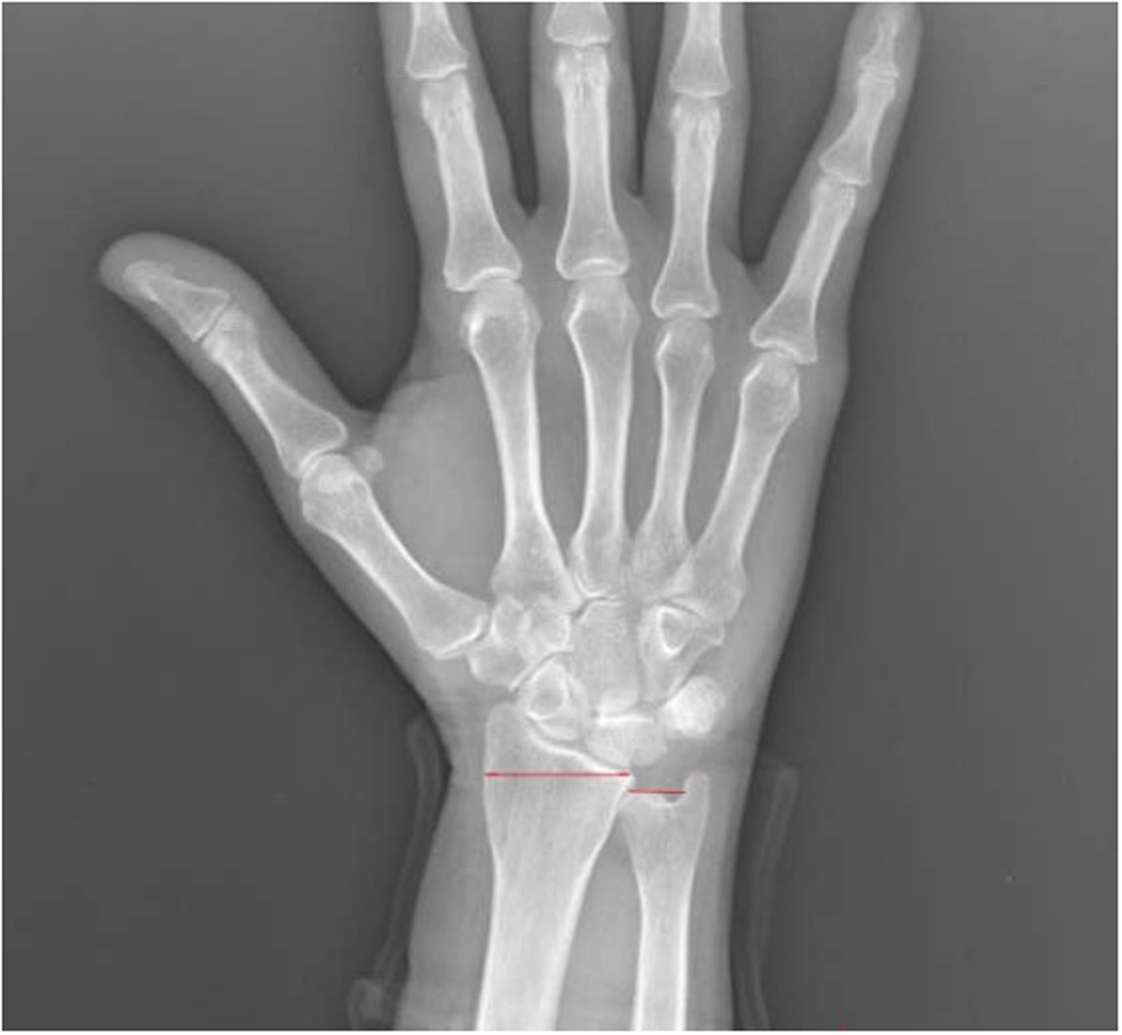
Negative Ulnar Variance

**Figure 2.**
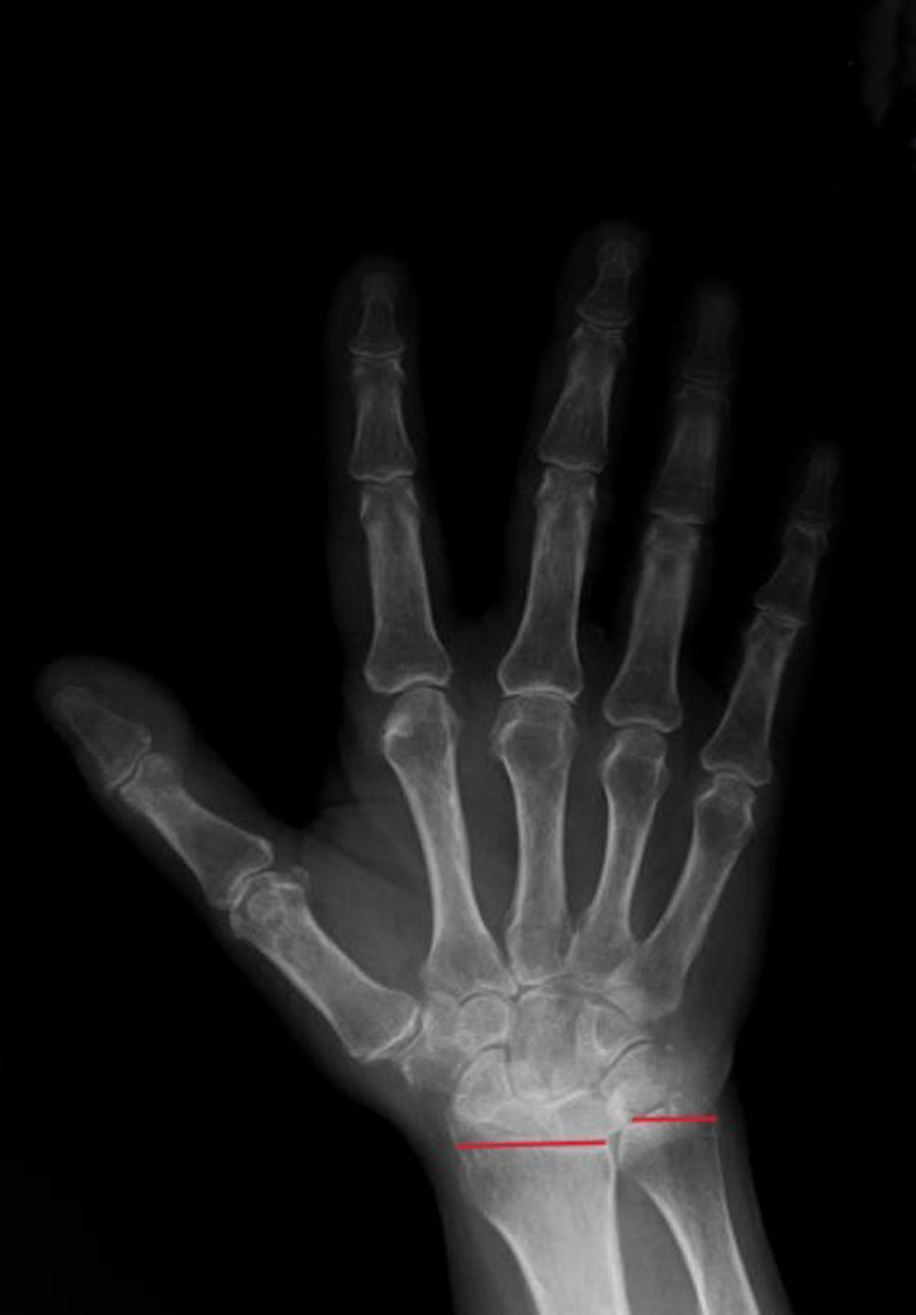
Positive Ulnar Variance

The radius and ulna are connected by the dorsal and volar radioulnar ligaments and are expected to be in a neutral position relative to each other within the wrist joint.^4^ Ulnar variance is a term used to describe the positional relationship between the distal ulna and radius. Negative ulnar variance is the presence of the ulna 1 mm or more proximal to the radius and has been associated with conditions such as ulnar impingement syndrome, scapholunate dissociation, and Kienböck’s disease (Figure 1). Positive ulnar variance is the presence of the ulna 1 mm or more distal to the radius and is associated with conditions such as ulnar impaction syndrome, cartilaginous tears in the carpal bones, early degenerative changes, and triangular fibrocartilaginous complex (TFCC) tear (Figure 2).^5^

In diseases such as RA, in which the wrist is frequently affected, disruption of the radioulnar relationship is possible. Unfortunately, however, there is no study in the literature examining ulnar variance in RA cases. Studies on this subject have generally focused on non-inflammatory diseases. The aim of this study was to determine the frequency of AUV and associated factors in RA patients.

## Materials and Methods

### Patients Selection

The study was performed on patients admitted to the Ondokuz Mayıs University Department of Rheumatology, a tertiary care center, for follow-up. Demographic, laboratory, and clinical data were recorded. Inclusion criteria included being classified as RA according to the 2010 ACR/EULAR RA classification criteria, being older than 18 years, having a disease duration > 5 years in terms of RA, having a history of arthritis in the wrist joints, and having a current X-ray image of both hands taken with the appropriate technique as described in Palmer’s article, with radiographs of the wrist in neutral forearm rotation, the elbow flexed at 90°, and the shoulder abducted at 90°.^6^ The exclusion criteria were radiocarpal joint ankylosis due to RA involvement, the presence of additional rheumatic diseases accompanying RA, a history of previous fracture or dislocation of the upper extremity, being involved in sports such as boxing, basketball, or volleyball, which may cause chronic wrist trauma, and having a profession requiring chronic heavy hand use.

### Assessments

Diagnostic latency was defined as the time interval between the onset of initial arthritis symptoms and the initiation of disease-modifying antirheumatic drug (DMARD) treatment. Knee or elbow involvement was characterized by a history or presence of chronic arthritis in these joints lasting for more than 3 months, or by radiological evidence of chronic arthritis, such as erosion or joint space narrowing.

Hand and wrist radiographs were evaluated blindly by 2 rheumatologists (MP-SK). Hand radiographs were analyzed according to the Modified Sharp Score (MSS) system.^7^ The presence of any erosion or joint space narrowing (JSN) was used as a criterion to define “RA-type joint involvement (RJI).” “Severe joint involvement (SJI)” was defined as the presence of erosion with a score of 3 or higher or JSN with a score of 4 or more according to the MSS. Patients were also evaluated for ankylosis of any hand joint. Ulnar variance, as described by Hulten, was determined by measuring the distance between horizontal lines drawn at the subchondral bone of the distal radius, just beneath the articular cartilage, and the most distal subchondral border of the ulnar head.^8^ A displacement of 1 mm or more of the ulna relative to the radius was defined as AUV. Radiographic evaluation was performed with full agreement; if there was disagreement between the readers, the X-ray was re-evaluated and an agreed final decision was made.

Rheumatoid factor (RF) was measured by the nephelometric method; >15 IU/mL was considered positive. Anti-cyclic citrullinated peptide (anti-CCP) antibody IgG was measured by enzyme-linked immunosorbent assay ( ELISA) and >5 U/mL was considered positive. Biologic and target-specific DMARDs were used in cases resistant to conventional synthetic DMARD therapy if the patient had high disease activity according to the DAS-28 score (DAS-28 score >5.1). Patients were divided into 2 groups according to the presence or absence of AUV, and demographic, laboratory, clinical, and treatment data were compared between these 2 groups.

### Statistical Method

Statistical analyses were conducted using SPSS 21.0 for Windows (IBM SPSS Corp.; Armonk, NY, USA). A power analysis conducted in advance indicated that the minimum sample size required for each group was 39. Descriptive statistics were expressed as mean ± SD, minimum-maximum values, frequency distributions, and percentages. The Kolmogorov-Smirnov test was applied to assess the normality of the quantitative data. For comparing clinical outcomes and demographic variables between the 2 groups, Student’s *t*-test, Mann–Whitney *U *test, and chi-square test were employed. Additionally, binary logistic regression analysis was utilized to evaluate whether age, biological drug usage, disease duration, gender, smoking status, RA-type, X-ray, presence of severe erosion, ankylosis, RF, and anti-CCP levels were linked with ulnar variance. A *P*-value below .05 was deemed statistically significant. Ethical approval for the study was granted by the Ethics Committee of Ondokuz Mayıs University Medical Faculty, under protocol number 2024/312, date 31.7.2024.

## Results

The study included 108 RA patients. Four patients with a history of trauma or fracture, 2 patients with complete ankylosis of the wrist, and 4 patients with incorrectly positioned radiographs were excluded. After the exclusion criteria, the remaining 98 RA patients were evaluated. The mean age was 58.11 ± 12.05 years (20-86) and 82% of the patients were female. The mean disease duration was 175.16 ± 100.5 (60-540) months, and the median diagnostic delay was 16.4 ± 11.18 (0-120) months. Rheumatoid factor and anti-CCP positivity were 67% and 71%, respectively. The rates of RJI, SJI, and ankylosis were 70%, 35%, and 26%, respectively. Abnormal ulnar variance was present in 47.9% of the cases. Three of the 47 patients with AUV had negative variance, while 44 had positive variance. In the group with AUV, the mean right hand ulnar variance measurement was +2.24 mm, while the mean left hand ulnar variance was +2.40 mm. When all RA patients were considered, the mean ulnar variance values were +1.06 mm for the right hand and +1.09 mm for the left hand. Table 1 shows demographic, laboratory, clinical, and treatment data.

RA patients with AUV+ had longer disease duration (*P* = .012), higher anti-CCP titer (*P* = .030), higher RF positivity (*P* = .021), more RJI (*P* < .001), SJI (*P* < .001), and ankylosis (*P* < .001) than the AUV− group. Also, the AUV+ group had a higher frequency of b/tsDMARD use (*P* = .012). Table 2 shows the comparison between AUV+ and AUV− groups.

In the multivariate analysis model, which included gender, disease duration, smoking status, RJI, SJI, ankylosis, RF, and anti-CCP, RJI (*P* = .003) and SJI (*P* = .018) were identified as independent predictive factors for AUV in patients with RA. The results of the binary logistic regression analysis are presented in Table 3.

## Discussion

To the best of the authors’ knowledge, this is the first study in the literature focusing on the evaluation of ulnar variance in RA patients. According to the findings, approximately 48% of RA patients had AUV and 93.6% (44/47) of these patients had positive ulnar variance. Disease duration, RF positivity, anti-CCP titer, RJI, SJI, and the presence of ankylosis were associated with AUV in RA patients. In addition, b/tsDMARD use was found to be higher in the AUV-positive group, and RJI and SJI were found to be independent predictive factors for AUV.

Ulnar variance defines the position of the ulna relative to the radius at the distal radioulnar joint, exhibiting increases during forearm pronation and decreases during supination.^9^ Consequently, the standard approach commonly used to demonstrate ulnar variance is a method described in detail in Palmer’s study, which involves obtaining an X-ray with the shoulder abducted to 90 degrees, the elbow flexed to 90°, and the hand in a neutral position.^10^ This method was used in the radiographs and evaluated the radiographs by measuring from the standard points defined by Hulten. Both methods have been shown to have high intraobserver and interobserver reliability in the literature.^9^ In studies conducted on cadavers to assess ulnar variance, X-ray imaging was compared with dissection following the use of computed tomography (CT) and magnetic resonance imaging (MRI) techniques, leading to the conclusion that X-ray imaging may be sufficient for detecting ulnar variance.^10^ Therefore, it was proposed that if routine X-ray imaging, commonly utilized in patients with RA, is performed using the Palmer method, both the disease findings and ulnar variance can be evaluated simultaneously without incurring additional costs.

Although there is currently no widely accepted study on ulnar variance in relation to the healthy population in Türkiye, 2 hospital-based studies have reviewed this topic. In one of these studies, the mean ulnar variance was found to be negative, with a mean value of −0.08 mm, while the other study reported a positive ulnar variance in 26% of patients.^11,12^ The fact that the data used in the studies were retrospective hospital data and the presence of diseases that necessitated hand radiography in the patients requires careful evaluation of whether these results accurately reflect the healthy population. In this study, unlike these 2 data, a significant positive ulnar variance pattern was found in RA patients. Positive ulnar variance may lead to loading problems on the ulnar side of the wrist and cause ulnar impaction syndrome, whereas negative ulnar variance may lead to progressive problems such as Kienböck’s disease with increased loading on the radial side of the wrist and subsequent degenerative changes.^13^ The most important cause of ulnar impaction syndrome is positive ulnar variance. It has been shown that a 2.5 mm advancement of the ulna towards the carpal bones causes up to a 42% increase in axial load on the surrounding ligaments and bones. This load may cause injuries and tears in the ulnar-sided stabilizing ligaments.^14^ The TFCC, lunate, and triquetral bones are the main structures affected.^15^ The clinical presentation typically includes pain aggravated by activities involving ulnar deviation or forearm pronation and may be accompanied by swelling after repetitive movements and a decreased range of motion in the wrist.^16^ These described findings can be easily confused with an arthritic exacerbation in the radiocarpal joint in a patient with known RA. In a significant proportion of RA patients, an acute phase response is not observed during exacerbation. This rate may reach up to 45% in the early stages of RA.^17^ This makes it more difficult to differentiate flares from degenerative pain. In patients with established RA experiencing wrist joint pain without an acute phase elevation, degenerative conditions such as ulnar impaction syndrome may contribute to the pain. For this reason, it was believed that secondary causes due to AUV should be kept in mind both in the differential diagnosis of arthritic flare-up and as a factor aggravating arthritis pain.

The wrist joint is one of the joints most commonly affected by RA.^18^ In RA wrist involvement, the formation of pannus with the proliferation of synovia leads to the destruction of the bone, ligament, joint capsule, and surrounding soft tissues. This destruction can result in malalignment of the distal radioulnar joint, manifesting as dorsal dislocation of the ulna, wrist supination over the radius, and volar subluxation of the extensor carpi ulnaris tendon.^19^ Pathologies affecting the distal radioulnar joint are known to be associated with AUV. Conditions such as Madelung and reverse Madelung deformities, distal radius/ulnar fractures, distal radioulnar joint injuries, TFCC tears, ulnar abutment syndrome, lunotriquetral ligament tears, Kienböck’s disease, and ulnar impingement syndrome are all associated with AUV.^20^ It is not surprising to find AUV in RA patients in whom the radioulnar joint is severely affected. Joint malalignment and deformities in RA are usually not seen in the early period and are late disease findings. For this reason, this study included patients with RA who had a disease duration of at least 5 years. The average disease duration is approximately 15 years when all patients participating in the study are considered. In the group exhibiting AUV, the mean disease duration was significantly longer than in the other group. These findings suggest that cumulative joint damage increases in RA patients over time, resulting in malalignment during the long-term progression of the disease. Studies in the literature have also shown that deformity and disability increase with disease duration in RA patients. In the review by Scott et al,^21^ it was reported that erosion developed in joints of RA patients at rates of up to 73% within the first 5 years. The patients in the AUV+ group had a mean disease duration of 4 years longer than the AUV- group. Additionally, AUV was found to be significantly more prevalent in patients with RJI, SJI, and ankylosis findings on X-ray examination. This indicates that impaired ulnar variance is likely a consequence of the increasing duration of the disease and the cumulative joint damage that occurs over time due to the progressive nature of RA.

Another significant factor in the occurrence of total joint damage in RA patients is the presence of poor prognosis markers. Rheumatoid factor is associated with more severe erosive disease and extra-articular manifestations in RA patients, and it has been shown to be associated with poor prognosis.^22^ Anti-CCP is an antibody associated with higher disease activity and more radiologic damage in RA patients, and anti-CCP antibody titers are correlated with radiologic damage.^23^ In this study, a relationship was found between RF positivity, high CCP titers, and AUV. It is noteworthy that anti-CCP antibodies can present at low titers, especially in early-stage RA patients, and may even become positive years before the clinical onset of the disease.^24^ A high anti-CCP titer is indicative of a more aggressive disease course, making it expected to observe more erosive and deforming disease in these patients. The high prevalence of anti-CCP positivity in the group with AUV may be linked to the greater deformity and more aggressive disease progression experienced by these patients. While both RF positivity and CCP positivity have been shown as a poor prognosis for RA, there is no study that directly correlates these biomarkers with wrist joint involvement. Some patients with RF and anti-CCP positivity, who are characterized as having a poor prognosis, may experience severe involvement in the knee joint, elbow joint, or proximal interphalangeal joints of the hand while exhibiting milder synovitis in the radiocarpal joints.^25^ Although morbidity and disability scores are high in these patients, some may show limited or no involvement of the wrist joint, with disease progression potentially affecting other joints instead, which may not significantly influence ulnar variance. The observed relationship between RF positivity and ulnar variance, but not anti-CCP positivity, in this study, may be attributed to this phenomenon. In this study, subgroups indicating which specific joints were affected and to what extent were not established. Moreover, the number of patients per subgroup in this study may not have provided sufficient statistical power to demonstrate this relationship.

In this study, another poor prognosis factor in RA patients was failure to respond to DMARD treatment.^26^ The fact that AUV was observed significantly more frequently in the group using biologic and target-specific DMARDs suggests that these patients have a more resistant course. According to EULAR recommendations, it is advised not to start biologic DMARDs without using methotrexate (MTX) in RA patients.^27^ In this country, starting a biologic DMARD is permissible if there is an inadequate response after at least 3 months of treatment with 3 conventional DMARDs, one of which must be MTX. This finding underscores the link between AUV and a more treatment-resistant disease course in RA. Given that the use of biologic and target-specific DMARDs is typically reserved for patients who have failed multiple conventional DMARDs, including methotrexate, it is evident that AUV is more prevalent in those with a poor prognosis and aggressive disease progression.

To further investigate the factors contributing to AUV, a regression analysis was conducted. These results indicated that the strongest associations with AUV were found in RJI and SJI, as observed on X-ray examination. In conclusion, it was believed that the higher prevalence of AUV in the patient cohort is driven by the combined effects of poor prognostic factors and prolonged disease duration, both of which contribute to greater joint damage.

One of the main limitations of this study is that the patients were diagnosed with RA at a single center and had long-standing disease. This limitation affects the generalizability of these findings. Further subgroup analyses may be necessary to elucidate the relationships between prognostic markers such as RF and anti-CCP positivity and AUV. For instance, joint involvement was not analyzed in specific subgroups, which may have resulted in the oversight of other potential associations. Since the radiologic methods employed to assess ulnar variance in the study were reliable, MRI was not utilized. Previous studies have demonstrated that X-ray examinations are comparable to MRI in detecting ulnar variance. The reliance on patient history and radiographic findings for defining knee and elbow involvement specifically may affect the reliability of this information and thus represent a limitation of the study.

Additionally, the limited number of patients included in the study, primarily due to the inclusion criteria, is another limitation. The aim was to include individuals with a disease duration of 5 years or more to observe potential joint damage resulting from RA. To minimize unnecessary radiation exposure, current X-rays were obtained from patients who were due for routine follow-up or who presented with acute wrist arthritis. In this clinic, joint damage is monitored in RA patients every 3 years through routine X-ray imaging, which further constrained the study population.

Despite these limitations, this study highlights that AUV may serve as an important clinical finding in RA patients with disease duration exceeding 5 years. Future larger-scale and prospective studies are warranted to clarify the significance of AUV in RA cases.

## Figures and Tables

**Table 1.** Comparison of Demographic Data of Patients According to Ulnar Variance

	Group 1 (n = 51) **Mean ± SD (range)**	Group 2 (n = 47) **Mean ± SD (range)**	*P*
Age (year)	57.15 ± 11.47 (24-80)	59.06 ± 12.45 (20-86)	.440
Disease duration (month)	150.58 ± 95.43 (60-540)	199.74 ± 111.92(60-480)	.012*
Age at onset of the symptoms (year)	44.62 ± 12.16 (10-72)	42.40 ± 13.25 (15-76)	.324
Diagnosis delay duration (month)	16.15 ± 12.17 (1-120)	16.51 ± 10.46 (0-120)	.931
Smoking (n%) No Yes	43 (84.3) 8 (15.7)	37 (78.7) 10 (21.3)	.475
Gender (n%) Female Male	43 (84.3) 8 (15.7)	37 (78.7) 10 (21.3)	.475
Biological drug use No Yes	15 (29.4) 36 (70.6)	5 (10.6) 42 (89.4)	.021*

Group 1: Normal Ulnar Variance. Group 2: Abnormal Ulnar Variance.

Anti-CCP, anti-cyclic citrullinated peptides; RA: rheumatoid arthritis; RF, rheumatoid factor.

*P < .05 significant.

**Table 2.** Comparison of Clinical Data of Patients According to Ulnar Variance

	Group 1 (n = 51) **Mean ± SD (range)**	Group 2 (n = 47) **Mean ± SD (range)**	*P*
RF titer	142.85 ± 100.93 (0-1780)	208.05 ± 199.72 (0-4030)	.245
Anti-CCP titer	248.19 ± 186.67 (0-3390)	476.04 ± 109.28 (0-7160)	.030*
RJI (n %) No Yes	28 (54.9) 23 (45.1)	1 (2.1) 46 (97.9)	.001*
SJI (n%) No Yes	48 (94.1) 3 (5.9)	15 (31.9) 32 (68.1)	.001*
Ankylosis (n%) No Yes	49 (96.1) 2 (3.9)	23 (48.9) 24 (51.1)	.001*
RF (n%) Negative Positive	22 (43.1) 29 (56.9)	10 (21.3) 37 (78.7)	.021*
Anti-CCP (n%) Negative Positive	18 (35.3) 33 (64.7)	10 (21.3) 37 (78.7)	.125
Lung involvement (n %) No Yes	49 (96.1) 2 (3.9)	43 (91.5) 4 (8.5)	.344
Elbow involvement (n %) No Yes	44 (86.3) 7 (13.7)	42 (89.4) 5 (10.6)	.641
Knee involvement (n%) No Yes	34 (66.7) 17 (33.3)	24 (51.1) 23 (48.9)	.116

Group 1: Normal Ulnar Variance. Group 2: Abnormal Ulnar Variance.

Anti-CCP, Anti-cyclic citrullinated peptides; RA, rheumatoid arthritis; RF, rheumatoid factor; RJI, RA-type joint involvement; SJI, severe joint involvement.

*P < .05 significant.

**Table 3.** Binary Logistic Regression Analysis with Abnormal Ulnar Variance as Dependent Variable and Age, Disease Duration, Gender, Smoking, RA-Type X-Ray, Severe Erosion, Ankylosis, Rheumatoid Factor, Anti-CCP, Biological Drug Use As Independent Variables

	B	Std. Error	Wald	*P*	Exp (B)	95% CI for Exp (B)	
						Lower	Upper
Disease duration	0.001	0.004	0.138	.711	0.999	0.991	1.006
Gender	−0.798	0.921	0.750	.386	0.450	0.074	2.739
Smoking	−1.407	0.988	2.029	.154	0.245	0.035	1.697
RJI	3.871	1.321	8.584	.003*	0.021	0.002	0.278
SJI	2.247	0.948	5.612	.018*	0.106	0.016	0.679
Ankylosis	1.322	1.142	1.339	.247	0.267	0.028	2.501
RF	0.892	0.956	0.869	.351	2.439	0.374	15.899
Anti-CCP	0.278	0.961	0.084	.772	1.321	0.201	8.679
Biological drug use	−2.123	1.340	2.508	.113	0.120	0.009	1.656

Anti-CCP, Anti-cyclic citrullinated peptides; RA, rheumatoid arthritis; RF, rheumatoid factor; RJI, RA-type joint involvement; SJI, severe joint involvement.

*P < .05 significant.

## Data Availability

The data that support the findings of this study are available on request from the corresponding author.
